# Association of Social Media Use With Mental Health Conditions of Nonpatients During the COVID-19 Outbreak: Insights from a National Survey Study

**DOI:** 10.2196/23696

**Published:** 2020-12-31

**Authors:** Bu Zhong, Zhibin Jiang, Wenjing Xie, Xuebing Qin

**Affiliations:** 1 Donald P Bellisario College of Communications Pennsylvania State University University Park, PA United States; 2 School of Journalism and Communication South China University of Technology Guangzhou China; 3 School of Journalism and Communication Shanghai International Studies University Shanghai China; 4 School of Communication and the Arts Marist College Poughkeepsie, NY United States; 5 School of Communication East China Normal University Shanghai China

**Keywords:** COVID-19, mental health, social media, health information support, secondary traumatic stress, vicarious trauma, social support

## Abstract

**Background:**

Considerable research has been devoted to examining the mental health conditions of patients with COVID-19 and medical staff attending to these patients during the COVID-19 pandemic. However, there are few insights concerning how the pandemic may take a toll on the mental health of the general population, and especially of nonpatients (ie, individuals who have not contracted COVID-19).

**Objective:**

This study aimed to investigate the association between social media use and mental health conditions in the general population based on a national representative sample during the peak of the COVID-19 outbreak in China.

**Methods:**

We formed a national representative sample (N=2185) comprising participants from 30 provinces across China, who were the first to experience the COVID-19 outbreak in the world. We administered a web-based survey to these participants to analyze social media use, health information support received via social media, and possible psychiatric disorders, including secondary traumatic stress (STS) and vicarious trauma (VT).

**Results:**

Social media use did not cause mental health issues, but it mediated the levels of traumatic emotions among nonpatients. Participants received health information support via social media, but excessive social media use led to elevated levels of stress (*β*=.175; *P*<.001), anxiety (*β*=.224; *P*<.001), depression (*β*=.201; *P*<.001), STS (*β*=.307; *P*<.001), and VT (*β*=.688; *P*<.001). Geographic location (or geolocation) and lockdown conditions also contributed to more instances of traumatic disorders. Participants living in big cities were more stressed than those living in rural areas (*P*=.02). Furthermore, participants from small cities or towns were more anxious (*P*=.01), stressed (*P*<.001), and depressed (*P*=.008) than those from rural areas. Obtaining more informational support (*β*=.165; *P*<.001) and emotional support (*β*=.144; *P*<.001) via social media increased their VT levels. Peer support received via social media increased both VT (*β*=.332; *P*<.001) and STS (*β*=.130; *P*<.001) levels. Moreover, geolocation moderated the relationships between emotional support on social media and VT (*F*_2_=3.549; *P*=.029) and the association between peer support and STS (*F*_2_=5.059; *P*=.006). Geolocation also interacted with health information support in predicting STS (*F*_2_=5.093; *P*=.006).

**Conclusions:**

COVID-19 has taken a severe toll on the mental health of the general population, including individuals who have no history of psychiatric disorders or coronavirus infection. This study contributes to the literature by establishing the association between social media use and psychiatric disorders among the general public during the COVID-19 outbreak. The study findings suggest that the causes of such psychiatric disorders are complex and multifactorial, and social media use is a potential factor. The findings also highlight the experiences of people in China and can help global citizens and health policymakers to mitigate the effects of psychiatric disorders during this and other public health crises, which should be regarded as a key component of a global pandemic response.

## Introduction

### Background

After COVID-19 hit the world, health care workers have been rushing to care for infected patients and save lives. In the race to contain the COVID-19 pandemic, it is important for health care providers to not ignore another big risk—the invisible toll on mental health among nonpatients. One of the key lessons we have learned from the COVID-19 pandemic is that living in this public health crisis is extremely stressful for everyone, including those who appear healthy and have not contracted the virus [1]. This was evident in China, where fear, worry, and anxiety about COVID-19 and its health risks were overwhelming as soon as the disease spread across the country [2]. Following the lockdown announced in Wuhan on January 23, 2020, other Chinese cities quickly restricted people from moving around. A mandatory lockdown had forced millions of Chinese to stay at home for weeks, and even months. Living in an isolated environment could make people feel unattached, worried, lonely, and even traumatized [3]. The strong emotions experienced by the Chinese during the initial months of the COVID-19 outbreak were almost unimaginable to the outside world until it spiraled into a global pandemic [2], resulting in millions of cases and hundreds and thousands of deaths worldwide*.*

As researchers in epidemiology, medicine, and public health worldwide are continuously researching medications, vaccines, and coping strategies for COVID-19, it is important to study how health information on social media and lockdown or quarantine situations may contribute to the toll on people’s mental health. Many studies addressing the impact of the pandemic on mental health focus on COVID-19 patients [4], who have shown, for instance, posttraumatic stress symptoms [5] or depression [6]. This study aimed to reveal a holistic picture by evaluating the effects of the COVID-19 pandemic on the mental health of nonpatients based on a national representative sample from China. Using a national representative sample is critical for such a study given the widespread urban-rural differences in China. The COVID-19 pandemic has had different effects across China’s cities, towns, and rural areas, as the local responses in these regions depended on a complex interplay of numerous social and economic factors. Hence, the toll on mental health on people in these regions could considerably vary depending on their geographic location (or geolocation) and other internal or external factors.

### Social Media Use and COVID-19

Considerable research has been devoted to examining the mental health of patients diagnosed with COVID-19 [5-7] and medical staff who cared for and treated these patients during the pandemic [8,9]. However, not much is known about the mental health conditions of nonpatients, who might experience varying degrees of psychiatric disorders due to COVID-19. Research shows that as people are consistently exposed to negative information about a crisis, their anxiety and depression levels could elevate for an extended period [10]. As COVID-19 spread in China, people began to use WeChat, China’s leading social media app, more frequently. By February 8, 2020, over 100 “mini programs” were added to WeChat to provide epidemic status information, and the app’s users grew by nearly 60% within 3 weeks [11]. The growing use of social media for crisis management has been well documented in the literature, and social media is considered a powerful tool to share health information related to pandemic risks [12,13].

However, there are controversies concerning the links between social media use and mental health. Studies have found that social media use may decrease satisfaction of life [14] and increase self-harm, suicidal ideation [15,16], psychological distress, depression, and anxiety [14]. Systematic reviews have shown that most of these studies are from Western countries, and a few studies are from Asian countries [14,15]. Insights from Asian countries may not only deepen the understanding of the relationship between social media use and mental health but also provide suggestions for education and policies [14]. People benefit from using social media in terms of promoting behavior change [17], obtaining health information support, and staying connected with others [2]; however, social media could also spread fear or misinformation about COVID-19, thereby causing harm to their mental health and psychological well-being [13]. Thus, more effort is merited to study how using social media to seek and share health information could have an impact on the users’ mental health during a health crisis.

### Informational, Emotional, and Peer Support

One of the main reasons people have been sharing health information on social media during the COVID-19 outbreak is the social support they gain from other users—a phenomenon that can be best explained by the uses and gratifications theory [18,19]. This theory holds that people use certain media content or platforms to gratify specific informational needs and demands; otherwise, they would no longer come back and use it again. Health information provides significant social support to people with health concerns, resulting in a number of benefits that help symptom control, disease recovery, life safety, and overall well-being [20]. Social support is defined as “the individual feeling valued and cared for by their social network as well as how well the person is embedded into a network of communication and social obligation” [21]. In other words, social support refers to the perception that one is cared for and support is exchanged through interpersonal interactions [22]. Helgeson [23] argues that social support has 3 main forms: informational, emotional, and instrumental support. Informational support refers to the provision of advice, guidance, and other useful information [24]. Emotional support exhibits the expressions of care, concern, empathy, and sympathy [23]. Instrumental support represents the concrete and direct ways in which people assist others [25,26]; this is referred to as “peer support” in this study.

The support users obtain on social media through accessing and sharing pandemic-related information can be viewed as health information support [2]. It functions like a type of social support received from family members, friends, colleagues, or peers during the pandemic. Ample research has identified that health information support provides patients with significant care and emotional support [27]. This type of support can also improve users’ capability of making informed medical decisions [28]. Identifying with social media groups has been found to increase one’s self-esteem and self-efficacy, and thus reduce uncertainty about the self [29]. Seeking support and social connection is a critical point in the lives of people with chronic conditions [30].

In addition to informational support, social media users gain emotional and peer support from the health information they access [2]. Emotional support is a key component of peer support in health care settings. High emotional support is known to mitigate the stress response and prevent consequent adverse effects on the progression of depression, posttraumatic stress disorder, and prostate cancer [31]. Patients who report more tangible emotional support are more likely to have experienced a positive social interaction with fellow patients and medical professionals [32]. WeChat users can obtain emotional support by chatting with friends, joining social media groups of their interests, and staying connected with others [20].

Peer support is a subcategory of social support, and it is differentiated by the source of support received from peers who are in a similar demographic group or illness community. Social media groups are important platforms to discuss medical conditions, share personal experiences, and seek health information [33,34]. In this study, peer support is defined as a type of support social media users receive from others when they share their knowledge and experiences of COVID-19. However, the quality of health information on the internet may vary depending on the sources, and health misinformation has become a severe threat to public health [35]. For instance, the inaccuracy of online health information deteriorates the physician-patient relationship and erodes trust in doctors [20]. Thus, it would be important to study how the social support about COVID-19 that people receive on social media is associated with their mental health and psychiatric disorders. Overall, peer support complements and enhances mental health by providing the necessary emotional, social, and practical assistance for managing disease and staying healthy.

### Mental Health and the Pandemic

Global health crises, especially the COVID-19 pandemic, have diverse and substantial health implications on the human society [1]. The deleterious consequences related to COVID-19 along with the unprecedented mitigation strategies pose major threats to the well-being and mental health of people worldwide [36]. COVID-19 has significantly changed various aspects of our routine life, including economics, travel, interpersonal communication, and health management [37]. When an individual’s routine life is suddenly and severely disrupted by a pandemic, the human brain may no longer function normally as usual; this can, consequently, lead to stress or psychiatric disorders [38]. This affects not only the people who have been diagnosed with COVID-19 but also those who seem to be “normal” or “healthy.” Thus, a psychiatric disorder may occur in individuals who have not been infected by the virus themselves but have experienced or witnessed the challenges of other individuals during the COVID-19 pandemic [39]. Although findings regarding the relationship between social support and mental health are inconsistent, social support, in general, has been found to provide physical and psychological advantages to combat stressful events and recover from psychological distress [40]. Lack of social support, however, has been linked with the onset and development of depression [41], mood disorders [42], and other medical illnesses such as multiple sclerosis and rheumatoid arthritis [43,44].

### Research Questions

Building upon previous findings, this study is among the first to investigate nonpatients’ mental health conditions such as stress, anxiety, depression, secondary traumatic stress (STS), and vicarious trauma (VT) during the peak of the COVID-19 epidemic in China. We measured both STS and VT, but not posttraumatic stress symptoms, as COVID-19 was an ongoing crisis when we collected data for analyses. Thereafter, we analyzed the internal (ie, demographics) and external (ie, pandemic and environmental conditions) factors that may contribute to possible psychiatric disorders such as STS and VT. Specifically, we investigated the following research questions (RQs):

- RQ1: Did nonpatients experience any stress, anxiety, depression, STS, or VT at the peak of the COVID-19 outbreak in China?
- RQ2: Did people living in different geolocations experience varying levels of psychiatric disorders?
- RQ3: How did demographics and pandemic situations, such as lockdown, quarantine conditions, and death numbers, contribute to possible psychiatric disorders?
- RQ4: How was social media use associated with psychiatric disorders?
- RQ5: How did health informational, emotional, and peer support mediate the relationship between demographics or pandemic situations and STS or VT?
- RQ6: Would geolocation interact with the health informational, emotional, and peer support people received through social media to predict psychiatric disorders?

## Methods

### Sampling

A marketing research company helped recruit a national representative sample for this study by using the quota sampling method, with a survey investigating how social media use affected the mental health conditions of people in China. In all, 4500 questionnaires were distributed, and 3820 individuals participated in this survey. After excluding incomplete data, responses from a total of 2185 participants were included for further analyses. These participants were from 30 provinces across China, and lived in big cities (eg, Beijing, Shanghai, Guangzhou, and Wuhan), small cities or towns, and rural areas. To avoid retraumatizing participants with questions about mental health issues or COVID-19, the survey comprised screening questions to exclude patients with COVID-19 or those who currently or previously had depressive or traumatic disorders.

After obtaining approval from the university’s institutional review board, we conducted a web-based survey in February 2020, which was the peak of the COVID-19 epidemic in China when most people were forced to live under lockdown conditions due to the spike of confirmed cases. For this survey, participants received a small financial incentive (¥10 or US $1.34 each) and were asked to answer specific questions related to demographics, social media use, changes in mental health conditions, and lockdown conditions. Participants who indicated interest in this study were sent a message that contained the survey URL and login credentials. The survey was password-protected and could not be accessed without these credentials.

### Measures

Stress, anxiety, and depression were measured using a 4-point Likert scale, with scores ranging from 0 (“Did not apply to me at all”) to 3 (“Applied to me very much, or most of the time”); all other measures were assessed using a 5-point Likert scale, with scores ranging from 1 (“strongly disagree”) to 5 (“strongly agree”).

#### Social Media Usage

WeChat is China’s dominant social media app, and participants’ use of this app was measured using a 6-item instrument that was originally developed for measuring Facebook addiction [45]. The wordings of the questionnaire items were slightly revised to better fit the participants’ actual WeChat use during the COVID-19 outbreak. For instance, participants were asked to rate how likely they agreed with 6 statements, including “You feel an urge to use WeChat more as you want to know more about the epidemic” and “You use WeChat for health information on the epidemic so much that it has had a negative impact on your life.” The scores on the 6 items were averaged to form the index of social media use (mean 3.123, SD 0.809; Cronbach α=.901). A higher value indicates excessive social media use.

#### Informational, Emotional, and Peer Support

The scales of informational, emotional, and peer support were adopted and revised based on previous studies [46,47]. Informational support was measured on the basis of 4 items, including “If I have a question or need help related to the coronavirus epidemic, I can usually find the answers on WeChat.” The scores on these 4 items were averaged to form the informational support index (mean 3.376, SD 0.900; Cronbach α*=*.868). Emotional support was measured on the basis of 4 items, including “The health information on WeChat helps me alleviate feelings of loneliness.” The emotional support index had a high level of internal consistency (mean 3.292, SD 0.892; Cronbach α=.908). Similarly, peer support was measured on the basis of 6 items, including, “WeChat friends give me additional information about the coronavirus epidemic that I am not familiar with.” The peer support index also had a high internal consistency (mean 3.245, SD 0.586; Cronbach α=.907).

#### Stress, Anxiety, and Depression

We used 7 items of the 21-item Depression Anxiety Stress Scale (DASS-21) [48] to measure stress, including “I felt that I was using a lot of nervous energy” and “I found myself getting agitated.” These questionnaire items were evaluated on a 4-point Likert scale, with scores ranging from 0 (“Did not apply to me at all”) to 3 (“Applied to me very much, or most of the time”). The sum of the scores on these 7 items formed the stress index (mean 4.968, SD 4.455; Cronbach α=.860). DASS-21 was used to measure both anxiety (mean 5.030, SD 4.799; Cronbach α=.860) and depression (mean 5.104, SD 4.975; Cronbach α=.860).

#### STS and VT

STS is the emotional distress a person experiences when they hear about first-hand trauma experiences of another person [49]. In this study, STS was measured using the 14-item instrument adopted from Bride et al [50], including “I felt emotionally numb” and “My heart started pounding when I thought about the coronavirus epidemic.” The scores on these 14 items were averaged to form the STS index (mean 2.466, SD 0.799; Cronbach α=.938). A higher value indicates higher level of STS.

The concept of VT was proposed by Pearlman and Saakvitne [51] in their description of the trauma experiences people have after being exposed to others’ trauma stories and having witnessed the pain, fear, and terror that traumatized survivors have endured. In this study, VT was measured using the 8-item instrument developed by Vrklevski and Franklin [52], including “I find myself thinking about distressing material at home” and “Sometimes I feel helpless to assist others in the way I would like.” The VT index had a good internal consistency (mean 3.349, SD 0.723; Cronbach α=.861).

Finally, pandemic situations were measured using the following questions: “How long is your residence area locked down to restrict entry of nonresidents?” “Do you have any family members or friends currently under quarantine?” “Do you know any family members or friends confirmed infected by coronavirus?” and “Do you know family members or friends who died due to the coronavirus epidemic?” Data on the participants’ age, gender, education, income, and geolocations were also obtained.

### Data Analyses

To answer RQ1, we first performed descriptive data analyses with anxiety, depression, stress, STS, and VT. Paired-sample *t* tests were used to compare the levels of different psychiatric disorders. To answer RQ2, we performed one-way analysis of variance with psychiatric disorders as the dependent variables and geolocation as the independent variable. For RQ3 and RQ4, we performed hierarchical regression analyses. STS was entered as the dependent variable in the model, followed by VT, stress, anxiety, and depression. Demographic information was entered in the first step of the model, and pandemic situations were entered in the second step, followed by social media use in the third step. For RQ5, we performed structural equation modeling. Demographics and pandemic situations were used as exogenous variables to predict informational support, emotional support, and peer support, which in turn predicted endogenous variables (ie, STS and VT). For RQ6, we used a generalized linear model to analyze the interaction between geolocation and informational, emotional, and peer support for predicting psychiatric disorders.

## Results

### Prevalence of Psychiatric Disorders

Compared with the latest census data published by the central government of China [53], the national sample attributes largely matched with the proportion of the Chinese population. The sample demographics were considered as internal factors that may influence the levels of stress, anxiety, or depression (Table 1). Pandemic situations and environmental conditions were considered as external factors that may contribute to STS or VT (Table 2).

The data showed that the pandemic had caused significant harm on people’s mental health. Only 3 weeks after a lockdown was announced in Wuhan, 10% (219/2185) of the participants reported they experienced moderate-to-severe anxiety, and 9.8% (215/2185) of the participants reported they experienced mild anxiety symptoms. Meanwhile, 5.5% (121/2185) of the national sample had moderate-to-severe depression, and 14.5% (316/ 2185) of the participants reported mild depression. These results are consistent with the findings from other surveys conducted in China during COVID-19 that report approximately 22% of the population experienced anxiety and 20% experienced a combination of depression and anxiety [3], although no study has evaluated traumatic disorders. This study shows that Chinese people displayed a moderate level of STS (mean 2.466, SD 0.799) and a relatively high level of VT (mean 3.934, SD 0.723), with significantly higher VT levels reported than STS levels (t_2184_=46.747; *P<*.001).

**Table 1:** Internal factors contributing to psychiatric disorders (N=2185).

Internal factor		Value
Age (years), mean (SD)		33.43 (31)
**Gender, n (%)**		2185 (100)
	Female	1192 (54.6)
	Male	993 (45.6)
**Income, n (%)**		2185 (100)
	Very low income	353 (16.2)
	Low income	445 (20.4)
	Medium income	1223 (56)
	High income	130 (5.9)
	Very high income	34 (1.6)
**Marital status, n (%)**		2185 (100)
	Unmarried	715 (32.7)
	Married	1470 (67.3)
**Education, n (%)**		2185 (100)
	Middle school or lower	246 (11.3)
	High school	492 (22.5)
	3-year college	587 (26.9)
	4-year college	766 (35.1)
	Graduate degree	94 (4.3)

**Table 2:** External factors contributing to psychiatric disorders (N=2185).

External factor		Value, n (%)
**Geolocation**		2185 (100)
	Rural area	592 (27.1)
	Small cities or towns	1189 (54.4)
	Big cities	404 (18.5)
**Lockdown time**		2185 (100)
	No lockdown	0 (0)
	1-2 weeks	1065 (48.7)
	3-4 weeks	795 (36.4)
	5-6 weeks	213 (9.7)
	7 weeks or more	112 (5.1)
**Known quarantine cases**		2185 (100)
	None	1983 (90.8)
	1 person	63 (2.9)
	2 people	73 (3.3)
	3 people	41 (1.9)
	4 or more people	25 (1.1)
**Known death cases**		2185 (100)
	None	2082 (95.3)
	1 person	31 (1.4)
	2 people	48 (2.2)
	3 people	18 (0.8)
	4 or more people	6 (0.3)
**Known infection cases**		2185 (100)
	None	2016 (92.3)
	1 person	73 (3.3)
	2 people	65 (3)
	3 people	24 (1.1)
	4 or more people	7 (0.3)

### Internal and External Factors Contributing to Psychiatric Disorders

Our analyses revealed that a range of internal (ie, demographics) and external (ie, pandemic and environmental conditions) factors are related to psychiatric disorders (Table 3), as well as stress, anxiety, and depression (Table 4). With regard to stress, participants who were younger (*β*=−.142; *P*<.001), male (*β*=.054; *P*=.04), married or divorced (*β*=.078; *P*<.001), and had a higher income (*β*=.049; *P*=.03) reported higher levels of stress than other participants during the pandemic. These demographics accounted for 2.3% of the variance in stress (*ΔR^2^*=.023; *P*<.001). Higher levels of stress were reported by participants who lived under lockdown for a longer time (*β*=.028; *P*=.04) and those who knew of more quarantine cases among their friends and family members (*β*=.105; *P*<.001), as well as more cases of COVID-19–related deaths (*β*=.117; *P*<.001). These pandemic situations accounted for 4.2% of the variance in stress (Δ*R*^2^=.042; *P*<.001).

With regard to anxiety, participants who were younger (*β*=−.085; *P*<.001), male (*β*=.058; *P*=.007), and married or divorced (*β*=.054; *P*=.03) reported higher levels of anxiety than did the other participants. These demographics accounted for 0.8% of the variance in anxiety (Δ*R*^2^=.008; *P*=.01). Participants who lived longer in a lockdown situation (*β*=.051; *P*=.02) and those who knew of more quarantine cases among family members and close friends (*β*=.092; *P*<.001) as well as more cases of deaths among them (*β*=.085; *P*=.001) reported higher levels of anxiety. These lockdown situations accounted for 3.9% of the variance in anxiety (Δ*R*^2^=.039; *P*<.001).

As for depression, younger participants reported being more depressive than older participants (*β*=−.094; *P*<.001). Participants who knew of more quarantine cases in family members and close friends (*β*=.107; *P*<.001) as well as more cases of deaths among them (*β*=.073; *P*=.02) reported higher levels of depression. These pandemic situations accounted for 3.7% of the variance in depression (Δ*R*^2^=.037; *P<*.001).

With regard to psychiatric disorders such as STS and VT, participants who were younger (*β*=−.099; *P*<.001), more educated (*β*=.093; *P*<.001), and married (*β*=.081; *P*<.001) were more likely to show STS symptoms. These demographics accounted for 1.7% of the variance in STS (Δ*R*^2^=.017; *P*<.001). As these participants knew of more quarantine cases among family members and close friends, they were more likely to develop STS (*β*=.053; *P*=.048). These pandemic situations accounted for 1.5% of the variance in STS (Δ*R*^2^=.015; *P*<.001). Moreover, female (*β*=−.059; *P*=.007) and more educated (*β*=.085; *P*<.001) participants experienced higher levels of VT. These demographics accounted for 1.8% of the variance in VT (Δ*R*^2^=.018; *P*<.001). Knowledge of quarantine cases among family members and close friends also positively predicted VT (*β*=.057; *P*=.003). These pandemic situations accounted for 0.6% of the variance in VT (Δ*R*^2^=.006; *P*=.01).

**Table 3:** Factors contributing to secondary traumatic stress and vicarious trauma.

Variables		Secondary traumatic stress									Vicarious trauma						
			*β*		*t* test (*df*)^a^		*P* value		Δ*R*^2^		*β*		*t* test (*df*)^a^		*P* value		Δ*R*^2^
**Step 1: Demographics**								.017^b^								.018^b^	
	Age		−.099		−*3.899 (5)*^*c*^		<.001		—^d^		−.013		−.502 (*5*)		.53		—
	Gender		.015		.673 (*5*)		.54		—		−.059		−*2.705 (5)*		.007		—
	Income		−.032		−1.365 (*5*)		.46		—		.045		1.918 (*5*)		.05		—
	Marriage		.081		*3.197* *(5)*		<.001		—		.046		1.810 (*5*)		.06		—
	Education		.093		*3.887* *(5)*		<.001		—		.085		*3.570* *(5)*		<.001		—
**Step 2: Pandemic situations**								.015^b^								.006^e^	
	Lockdown time		.041		1.865 (*4*)		.07		—		.015		.666 (*4*)		.54		—
	Known quarantine case		.053		*1.976* *(4)*		.048		—		.057		*2.122* *(4)*		.003		—
	Known infected cases		.022		.732 (*4*)		.46		—		−.026		−.839 (*4*)		.40		—
	Known death cases		.052		1.783 (*4*)		.095		—		−.006		−.188 (*4*)		.79		—
**Step 3: Social media use**								.091^b^								.479^b^	
	Social media use		.307		*14.899 (1)*		<.001		—		.688		*43.315* *(1)*		<.001		—

^a^Two-tailed *t* tests were performed.

^b^This denotes *P*<.001.

^c^Italiziced values indicate statistical significance.

^d^Not applicable.

^e^This denotes *P*<.005.

**Table 4:** Factors contributing to stress, anxiety, and depression.

Variables		Stress										Anxiety									Depression						
			*β*		*t* test (*df*)^a^		*P* value		Δ*R*^2^		*β*			*t* test (*df*)^a^		*P* value		Δ*R*^2^		*β*			*t* test (*df*)^a^		*P* value		Δ*R*^2^
**Step 1 Demographics**								.023^b^									.008^c^									.007^c^	
	Age		−.142		−*5.452 (5)*^*d*^		<.001		—^e^		−.085			−*3.316 (5)*		<.001		—		−.094			−*3.678 (5)*		<.001		—
	Gender		.054		*2.445 (5)*		.04		—		.058			*2.650* *(5)*		.007		—		.031			1.419 (*5*)		.147		—
	Income		.049		*2.022 (5)*		.03		—		.017			.729 (*5*)		.459		—		.019			.782 (*5*)		.423		—
	Married		.078		3.035 (*5*)		*<.001*		—		.054			*2.412* *(5)*		.03		—		.049			1.923 (*5*)		.125		—
	Education		.035		1.424 (*5*)		.14		—		.005			.210 (*5*)		.806		—		−.003			−.120 (*5*)		.930		—
**Step 2 Pandemic situations**								.042^b^									.039^b^									.037^b^	
	Lockdown time		.028		*2.438 (4)*		.04		—		.051			*2.329* *(4)*		.02		—		.035			1.609 (*4*)		.114		—
	Known quarantine cases		.105		*3.070 (4)*		<.001		—		.092			*3.478* *(4)*		<.001		—		.107			*4.045* *(4)*		<.001		—
	Known infected cases		.006		1.256 (*4*)		.623		—		.037			1.217 (*4*)		.223		—		.036			1.168 (*4*)		.242		—
	Known death cases		.117		*3.887 (4)*		<.001		—		.085			*2.901* *(4)*		.001		—		.073			*2.483* *(4)*		.02		—
**Step 3 Social media use**								.030^b^									.048^b^									.039^b^	
	Use of WeChat		.175		*8.175 (1)*		<.001		—		.224			*10.672* *(1)*		<.001		—		.201			*9.521* *(1)*		<.001		—

^a^Two-tailed *t* tests were performed.

^b^This denotes *P*<.001.

^c^This denotes *P*<.01.

^d^Italiziced values indicate statistical significance.

^e^Not applicable.

### Geolocation and Psychiatric Disorders

Participants living in big cities, small cities or towns, and rural areas reported varying levels of stress (*F*_2,2075_=7.224; *P*<.001). After COVID-19 hit China, participants living in big cities (mean 5.036, SD 4.518) were more stressed than those living in rural areas (mean 4.367, SD 4.351; *P*=.02). Participants living in small cities or towns (mean 5.238, SD 4.460) were also more stressed than those living in rural areas (*P*<.001), but the difference was small between participants living in big cities and those living small cities or towns. A similar pattern was also observed for anxiety, as participants living in these three geolocations reported varying levels of anxiety (*F*_2,2183_=3.569; *P*=.03). Participants from small cities or towns (mean 5.270, SD 4.747) were more anxious than those from rural areas (mean 4.647, SD 4.820; *P*=.01), but no difference was found between participants from big cities (mean 4.883, SD 4.891) and those from small cities or towns (*P*=.16). Moreover, no significant difference was found between participants from big cities and those from rural areas (*P*=.45).

A similar pattern was observed for depression, concerning the impact of geolocations (*F*_2,2183_=3.569; *P*=.03). Participants from small cities or towns (mean 5.344, SD 4.877) experienced more depression than those from rural areas (mean 4.682, SD 5.092; *P*=.008). No significant difference was observed between participants living in small and big cities (mean 5.015, SD 5.054; *P*=.25) nor between participants living in big cities and rural areas (*P*=.30).

### Social Media Use and Psychiatric Disorders

More social media use contributed to STS (*β*=.307; *P*<.001; Δ*R*^2^=.091; sum of *R^2^*=.124) and VT (*β*=.688; *P*<.001; Δ*R*^2^=.479; sum of *R^2^*=.481), as shown in Table 3. Participants who used more social media also reported higher levels of stress (*β*=.175; *P*<.001; Δ*R*^2^=.030; sum of *R^2^*=.095), anxiety (*β*=.224; *P*<.001; Δ*R*^2^=048; sum of *R^2^*=.095), and depression (*β*=.201; *P*<.001; Δ*R*^2^=.039; sum of *R^2^*=.083).

### Mediating Effects of Informational, Emotional, and Peer Support

**Figure 1:**
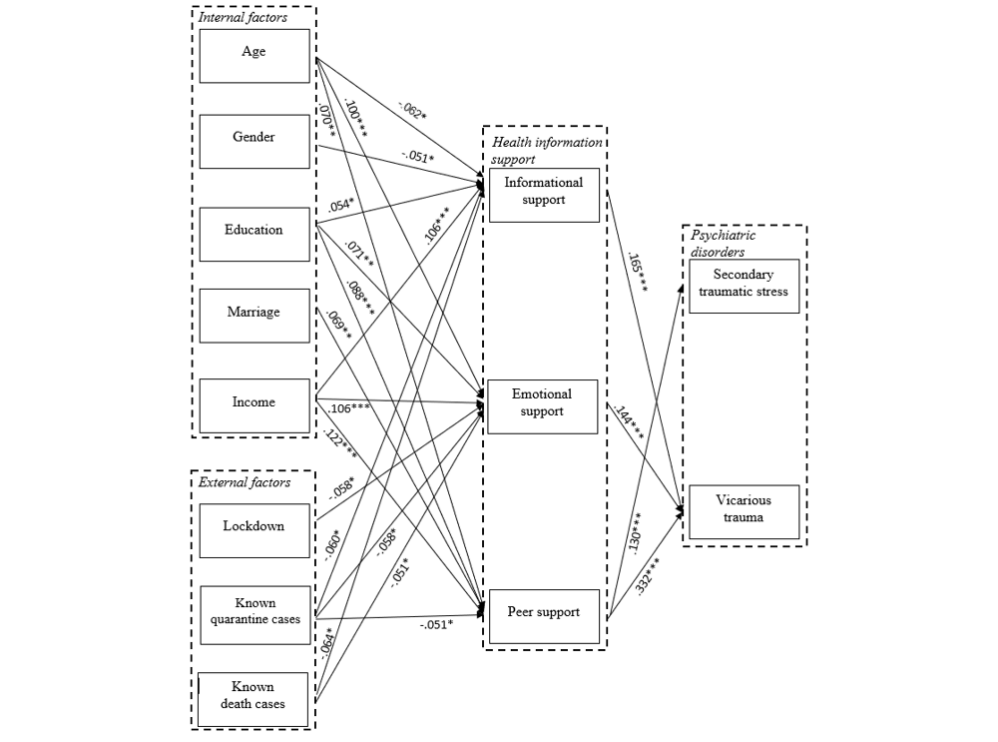
Structural equation modeling to predict secondary traumatic stress and vicarious trauma among participants during the COVID-19 outbreak in China.

Finally, structural equation modeling was performed to evaluate the mediating effects of informational, emotional, and peer support that the participants gained from health information shared on social media. The model had a good fit as demonstrated by the following indices: *χ^2^*_19_=25.286, minimum discrepancy divided by its *df* (CMIN/*df*)=1.331, *P*=.15, root mean square error of approximation=0.012, comparative fit index=.999, and Bentler-Bonett Normed fit index=0.996. Figure 1 shows that participants who were younger (*β*=−.062; *P*=.03), female (*β*=−.051; *P*=.05), and more educated (*β*=.054; *P*=.05) and those who earned a higher income (*β*=.106; *P*<.001) received more informational support on social media. On the other hand, participants who were older (*β*=.100; *P*<.001) and more educated (*β*=.071; *P*=.007) and those who had a higher income (*β*=.106; *P*<.001) received more emotional support in using social media. Participants who were older (*β*=.070; *P*=.005), married (*β*=.069; *P*=.004), and more educated (*β*=.088; *P*<.001) and those who had a higher income (*β*=.122; *P*<.001) received more peer support in using social media.

Knowledge of more quarantine cases among family members and close friends could negatively affect informational support (*β*=−.060; *P*=.04), emotional support (*β*=−.058; *P*=.03), or peer support (*β*=−.051; *P*=.02). Living for longer periods in a lockdown environment resulted in less emotional support via social media use (*β*=−.058; *P*=.03). More knowledge of deaths due to COVID-19 among family members and friends resulted in less informational support (*β*=−.064; *P*=.05) and emotional support (*β*=−.051; *P*=.03) via using social media. Both informational support (*β*=.165; *P*<.001) and peer support (*β*=.332; *P*<.001) were found to be associated with higher reported levels of VT. More peer support also increased levels of STS (*β*=.130; *P*<.001), whereas more emotional support led to an increase in VT levels (*β*=.144; *P*<.001), but not STS levels (*P*=.36).

### Moderating Role of Geolocation

**Figure 2:**
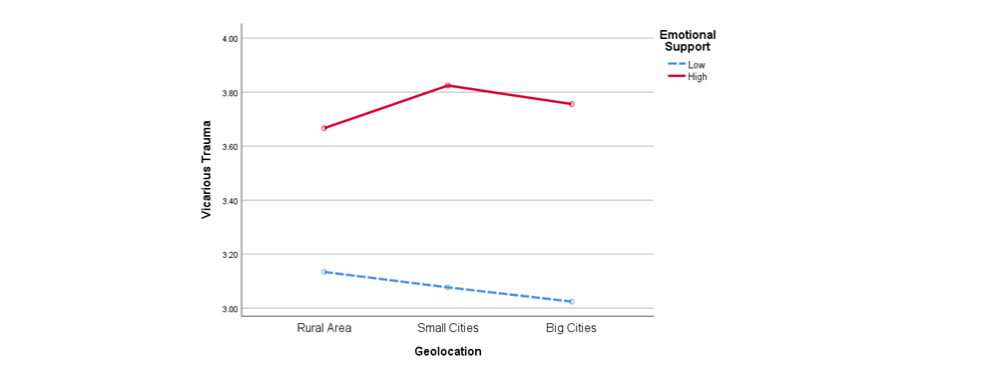
Interaction between geolocation and emotional support for vicarious trauma.

Geolocation had an interaction with emotional support in predicting VT (*F*_2_=3.549; *P*=.029; see Figure 2). Participants from rural areas reported higher levels of VT when they received higher emotional support via social media (mean 3.666, SD 0.796) than when they received lower emotional support (mean 3.134, SD 0.606; t_462_=7.947; *P*<.001); however, the differences were even larger for participants from small cities or towns (high emotional support: mean 3.825, SD 0.761 vs low emotional support: mean 3.077, SD 0.628; t_913_=16.012; *P*<.001) and those from big cities (high emotional support: mean 3.756, SD 0.659 vs low emotional support: mean 3.024, SD 0.764; t_307_=8.713; *P*<.001).

**Figure 3:**
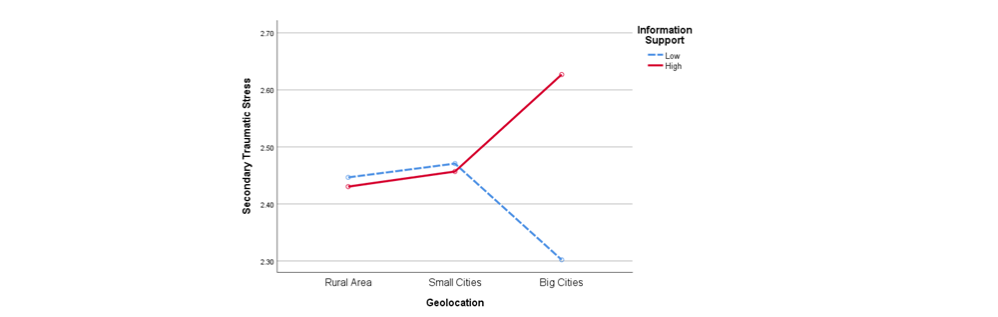
Interaction between geolocation and information support for secondary traumatic stress.

As shown in Figure 3, geolocation interacted with information support in predicting STS (*F*_2_=5.093; *P*=.006). For participants who lived in rural areas or small cities, no significant difference in STS levels was found between those who received more information support and those who received relatively less information support via social media. However, among the participants who lived in big cities, those who received more information support via social media reported higher levels of STS (mean 2.627, SD 0.953) than those who received relatively less information support on social media (mean 2.302, SD 0.802; t_299_=3.210; *P*=.001).

**Figure 4:**
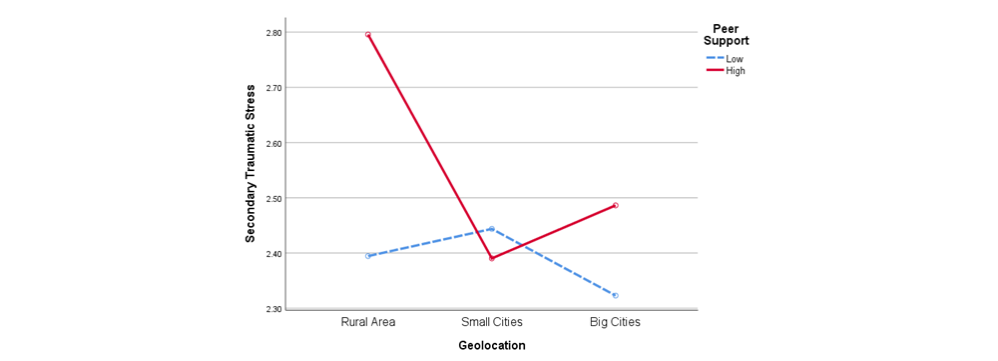
Interaction between geolocation and peer support for secondary traumatic stress.

As shown in Figure 4, geolocation also moderated the relationship between peer support and STS (*F*_2_=5.059; *P*=.006). For participants who lived in rural areas, those receiving more peer support experienced higher levels of STS (mean 2.796, SD 1.029) than those receiving less peer support (mean 2.395, SD 0.732; t_350_=3.369; *P*=.001). However, for participants who lived in big and small cities, peer support on social media did not make a difference to the level of STS.

## Discussion

### Principal Findings

As the ongoing COVID-19 pandemic affects people worldwide, it is crucial for researchers to conduct investigations that address the mental health consequences of the pandemic in order to mitigate the invisible harms caused to the general population. The findings of this study suggest that the mental health effects of the COVID-19 pandemic could have complex and multifactorial causes, including biological, behavioral, and environmental determinants such as social media usage. The analyses show that COVID-19 had taken a severe toll on the mental health of nonpatients, almost as soon as it started to spread in China. Social media use in addition to the lockdown environment and quarantine conditions have contributed the most to the overall toll on mental health.

Moreover, the analyses suggest that severe psychiatric disorders emerged in the general population of China, with 20% of the participants reporting anxiety, including 1 in 10 participants reporting moderate-to-severe anxiety. Furthermore, approximately 20% of the national sample reported depressive symptoms, of which 5.5% had moderate-to-severe depression. Overall, moderate levels of STS and considerably higher levels of VT were reported by these Chinese participants. The prevalence of psychiatric disorders reported seems particularly detrimental when we consider the facts that none of the participants were diagnosed with COVID-19 at the time of participation or had history of any psychiatric disorders before the COVID-19 outbreak.

Our findings suggest that a number of internal and external factors are related to stress, anxiety, and depression. Among the internal factors, participants who were younger, male, and more educated were more stressful or anxious than other participants at the peak of the COVID-19 outbreak in China. This finding is not consistent with previous studies in a nonpandemic context, which report that female participants tended to be more stressful. A possible explanation may be that younger individuals and men tended to go out more often to help others buy groceries than did older individuals or women, who spend more time at home in a lockdown situation [20]. Another explanation may be that compared to women, men are more susceptible to be stressed because of health reasons [51]. Previous studies have also shown that younger adults had a higher rate of psychological distress than older adults [54]. Future research should also examine different stress types and resilience mechanisms in relation to gender and age to understand how different stressors affect mental health differently.

The finding that social media use contributes to psychiatric disorders could be attributed to the fact that younger people relied more on social media and received more information about the pandemic through social media. It is worth noting that participants with higher income reported being more stressful and anxious than those who were less affluent. Younger, married, and more educated participants also reported having more STS symptoms, with no gender difference observed, suggesting that STS affected both men and women similarly. However, younger and more educated female participants reported higher levels of VT, which requires further research on why VT could affect women more than men.

Among the external factors, geolocation was found to be a critical determinant of mental health in COVID-19 nonpatients. In general, participants living in big cities felt similar levels of anxiety and depression as those living in small cities or towns, whereas participants in rural areas were the least stressful, anxious, and depressive. This may be due to the living conditions of big cities, which are more densely populated, and participants thus saw more infected patients and knew of more COVID-19–related deaths. In China, the medical resources are more readily available in cities than in rural areas; however, availability of more resources did not lead to less anxiety or fear among urban residents. This finding indicates that people in China had accurately assessed the severity of the emerging COVID-19 epidemic in the initial weeks.

Our findings suggest that even the individuals who were not themselves infected with COVID-19 nor were in quarantine could have experienced stress, anxiety, or depression as long as they lived in a lockdown situation or witnessed cases of infection, quarantine, and deaths among their family members or friends. The COVID-19 pandemic did not only endanger those infected with the virus but had also pushed China’s general population into a mental health crisis, and many people even reported that they experienced emotional trauma. All these occurrences were reported within 2 months of the COVID-19 outbreak. Thus, it is evident that living in the pandemic environment can be very debilitating and, in many cases, it has devastating effects on people’s psychological well-being with potentially lifelong consequences.

Another important finding of our study is the relationship between social media use for accessing health information and susceptibility of psychiatric disorders. This finding is consistent with those from a recent study in Wuhan, China—the first epicenter of COVID-19 globally, wherein researchers revealed that excessive social media use may lead to mental health issues [2]. In the present study, participants reported receiving social support from the health information shared on social media; this was especially true for those with more education and higher income. However, some differences were observed in terms of the specific informational, emotional, or peer support they received. Younger, female participants received the most informational support, whereas older participants received more emotional support than others, and participants who were married received more peer support than others. These results suggest that people process health information on social media differently and receive different types of support.

It is important to note that the approach does not suggest that social media use caused psychiatric disorders. We believe that an in-depth knowledge of social media use may contribute to a better understanding of the mechanisms of mental health conditions in a pandemic context.

On the other hand, knowledge of more quarantine cases during the outbreak was found to disrupt all the 3 types of support. Living longer in a lockdown environment had a similar effect of decreasing emotional support from social media use. Knowledge of more cases of deaths among family members or friends led to them reporting less informational and emotional support. Meanwhile, participants who gained more informational and peer support reported lower levels of STS and VT. When people received more emotional support by using social medial, they tended to report higher levels of VT, but not STS. These findings call for more research on the pathological effects of STS and VT on the nonpatients in a pandemic environment. 

It is noteworthy that although participants who knew of more quarantine cases reported receiving less informational, emotional, and peer support via social media, they still reported higher levels of STS and were more affected by mental illness. A possible explanation for this could be that participants who knew of more quarantine cases may have tried to seek information from other sources such as family, colleagues, and friends instead of via social media, and information from these sources may have increased their STS levels. Moreover, our additional data analyses showed that, for participants who knew of fewer than 4 quarantine cases, the information support (*r*=.069; *P*=.001), emotional support (*r*=.061; *P*=.005), and peer support (*r*=.113; *P*<.001) they received on WeChat were positively correlated to their STS levels. However, for participants who knew 4 or more quarantine cases, information support (*P*=.464), emotional support (*P*=.805), and peer support (*P*=.576) were no longer related to STS levels, suggesting that this group of participants generally maintained a high level of STS regardless of social media use. This finding is consistent with previous studies that described the potential anhedonia symptoms people experienced during COVID-19, which is characterized by the failure of experiencing pleasure from activities and is associated with depression, suicide, and other mental health issues [54].

### Conclusion

The results of this study suggest that the general public is extremely vulnerable to mental health issues during the COVID-19 pandemic. Living in a pandemic situation can have serious mental health consequences even for those without any history of psychiatric disorders or not being infected with COVID-19 themselves. A range of internal and external factors have been identified that likely contribute to these mental health conditions. For instance, age, gender, marital status, education, and income levels play an important role in individuals’ mental health conditions during the pandemic. Geolocation, lockdown duration, and social media use are also found to have an effect on mental health and traumatic disorders. Although people received health information support by using social media, excessive use of social media was found to be linked with elevated stress levels or psychiatric disorders. This finding does not suggest that social media use caused mental health issues but that it can mediate the levels of traumatic emotions experienced by people in the health crisis.

As the world is battling the COVID-19 pandemic, its detrimental effects on mental health will be more evident in the coming months and even after the pandemic is over. Health care providers thus need to carefully monitor psychosocial needs of the public and provide timely psychosocial support whenever needed. We believe that the findings in this research can help global citizens and health policy makers to mitigate psychiatric disorders in this as well as other public health crises, which should henceforth be regarded as a key component of general pandemic response.

## Abbreviations

DASS-21: 21-item Depression Anxiety Stress Scale
RQ: research question
STS: secondary traumatic stress
VT: vicarious trauma
